# Chasing Protection in Parkinson’s Disease: Does Exercise Reduce Risk and Progression?

**DOI:** 10.3389/fnagi.2020.00186

**Published:** 2020-06-19

**Authors:** Grace F. Crotty, Michael A. Schwarzschild

**Affiliations:** Department of Neurology, Massachusetts General Hospital, Boston, MA, United States

**Keywords:** Parkinson’s disease, exercise, physical activity, epidemiology, neuroprotection, disease modification, trials

## Abstract

Exercise may be the most commonly offered yet least consistently followed therapeutic advice for people with Parkinson’s disease (PD). Epidemiological studies of prospectively followed cohorts have shown a lower risk for later developing PD in healthy people who report moderate to high levels of physical activity, and slower rates of motor and non-motor symptom progression in people with PD who report higher baseline physical activity. In animal models of PD, exercise can reduce inflammation, decrease α-synuclein expression, reduce mitochondrial dysfunction, and increase neurotrophic growth factor expression. Randomized controlled trials of exercise in PD have provided clear evidence for short-term benefits on many PD measurements scales, ranging from disease severity to quality of life. In this review, we present these convergent epidemiological and laboratory data with particular attention to translationally relevant features of exercise (e.g., intensity requirements, gender differences, and associated biomarkers). In the context of these findings we will discuss clinical trial experience, design challenges, and emerging opportunities for determining whether exercise can prevent PD or slow its long-term progression.

## Introduction

Parkinson’s disease (PD) is the second most common neurodegenerative disorder and has an annual incidence rate of approximately 1 in 600 people aged 65 years or older (Hirtz et al., 2007; Pringsheim et al., 2014). The worldwide prevalence of PD is projected to rise from 6.2 million in 2015 to over 12 million people by the year 2040 (Dorsey and Bloem, 2018). The cause of PD is currently unknown but thought to be a consequence of genetic and environmental influences. Certain environmental factors (pesticides, dairy products, and traumatic brain injury) have been linked to increased risk of developing PD, whereas others have been linked to decreased risk (smoking, caffeine, urate, and physical activity; Ascherio and Schwarzschild, 2016). Currently, there are no disease-modifying therapies to prevent or slow down the neurodegenerative process (via neuroprotection) let alone reverse it (via neuro-restoration). Available therapies are symptomatic and act to improve the symptoms related to the dopamine deficit in nigro-striatal neurons. Exercise and physical activity have been proposed as interventions that could both decrease the risk of developing PD and modify the course of the disease. In this review we will define exercise, summarize some of the human data supporting exercise’s role in risk and progression of PD, review *in vitro* and *in vivo* studies demonstrating its neuroprotective properties, and conclude with evaluation of recent trials and a discussion of some of the challenges and opportunities for designing future exercise trials in PD.

“Physical activity” and “exercise” are often used interchangeably but are distinct concepts (Caspersen et al., 1985). Physical activity is a broader concept and refers to any bodily movement produced by skeletal muscles that results in expenditure of energy and includes both structured and unstructured activity (Bangsbo et al., 2019). It covers activities in the household, at work, during leisure, and sports. Exercise is a subset of physical activity and is defined as planned, structured, repetitive physical activity with a goal of greater well-being (Caspersen et al., 1985). Exercise programs typically comprise four core elements: aerobic and endurance training, strength training, flexibility training, and balance (Ellis and Rochester, 2018). Being physically active confers benefits for all aspects of health: increasing life expectancy; reducing the number of deaths; and lowering the risk of developing some of the major non-communicable diseases: coronary heart disease, type 2 diabetes, strokes, breast, and colon cancers (Lee et al., 2012).

The United States Department of Health and Human Services, Office of Disease Prevention and Health Promotion, and the World Health Organization recommend that individuals older than 65 years of age should do “at least 150 min of moderate-intensity aerobic physical activity throughout the week or at least 75 min of vigorous-intensity aerobic physical activity throughout the week or an equivalent combination of moderate- and vigorous-intensity activity (WHO, n.d.).” Moderate-intensity exercises includes brisk walking at 2.5 miles per hour, cycling less than 10 miles per hour, and playing doubles tennis. Vigorous-intensity activity consists of running, swimming laps, hiking uphill, and cycling greater than 10 miles per hour (American Heart Association, n.d.).

Despite these recommendations, approximately 33% of adults worldwide do not meet these recommended guidelines for physical activity (Hallal et al., 2012). Subjects with PD exercise even less than the general population (van Nimwegen et al., 2011), with less than a third of subjects walking at least 30 min per day (Lord et al., 2013). A cross-sectional review of exercise levels in 4,866 subjects with early PD (mean disease duration 5.5 years) revealed that 56% participated in either low levels of exercise (less than 150 min) or no exercise per week (Oguh et al., 2014). Over time, people with PD become less physically active, and do so at a rate greater than in age-matched healthy controls (Cavanaugh et al., 2015; Amara et al., 2019). For PD patients, increased or optimized exercise is a nearly universal advice provided by clinicians and PD advocacy organizations. In 2006, the American Academy of Neurology introduced both the counseling and monitoring of physical activity as a quality metric for PD care (Factor et al., 2016).

## Exercise and the Risk of Developing PD

Most prospective cohort studies have found an inverse relationship or trend between physical activity and the subsequent development of PD (LaHue et al., 2016), with a pooled hazard ratio of 0.66 for developing PD (95% CI 0.57–0.78) when comparing the highest level of physical activity with the lowest level (Yang et al., 2015). The first published study linking prior physical activity to PD was by Sasco et al. (1992) who carried out a case-control study in 50,002 male students attending the University of Pennsylvania and Harvard College between the years 1916 and 1950. Their study observed lower odds of developing PD if the student played varsity sports or underwent regular exercise in college with OR of 0.64 and 0.83, respectively. They also observed that in adulthood, those subjects who engaged in moderate exercise or heavy sports had lower odds of developing PD, although neither of these associations achieved statistical significance (Sasco et al., 1992).

Yang et al. (2015) performed a prospective cohort study of physical activity and the incidence of PD over an average of 12.6 years of follow-up in 43,368 subjects enrolled in the Swedish National March Cohort. They observed that subjects who reported more than 6 h per week of household and commuting activity had a 43% lower risk of developing PD compared to those who reported less than 2 h per week. Among men, the risk of developing PD was nearly halved for those reporting high vs low levels of general physical activity at baseline (HR: 0.53, 95% CI: 0.33–0.85). In women, no association between physical activity and PD development was observed. They also found no association for leisure time or occupational physical activity and PD, overall and in both genders separately (Yang et al., 2015). This lack of an association between occupational physical activity and developing PD has been seen in other studies (Shih et al., 2016).

Chen et al. assessed the relative risk for developing PD depending on the baseline level of physical activity in 48,574 men and 77,254 women from two prospective cohort studies, the Health Professionals Follow-Up Study (HPFS), and the Nurses’ Health Study (NHS), respectively. The baseline physical activity was recorded from the 1986 questionnaires and the presence of PD was assessed on biennial questionnaires from 1988 in HPFS and from 1994 in NHS. In the HPFS, men had a 30% lower risk of developing PD when comparing those in the highest quintile of physical activity compared to those in the lowest quintile (RR = 0.7; 95% CI 0.5–1.1). A 50% lower risk was seen when a 10-year lag was used between recording of baseline physical activity and development of PD, indicating that the reduced risk is unlikely to be as a result of prodromal PD motor symptoms. The study also found that men who spent 10 months or greater per year in vigorous activity in school, college, or between the ages of 30 to 40 years had a 60% lower risk of developing PD in later life compared to those participants who spent 2 months or less per year (RR = 0.4; 95% CI 0.2–0.7). However, the NHS failed to detect an inverse association in women for physical activity (total or vigorous) and the future risk of developing PD (Chen et al., 2005).

Other epidemiological studies that support the inverse relationship between physical activity and developing PD include studies from the NIH-AARP Diet and Health Study cohort (Xu et al., 2010), the Cancer Prevention Study II Nutrition Cohort (Thacker et al., 2008); the Finnish Mobile Clinic Health Examination Survey (Sääksjärvi et al., 2014), a populated-based case-control study in California (Shih et al., 2016), and the Survey of Health, Aging and Retirement in Europe (SHARE; Marques et al., 2018).

However, not all studies are supportive of these findings (Fertl et al., 1993; Logroscino et al., 2006; Roos et al., 2018). Logroscino et al. (2006) carried out a prospective cohort study of 10,114 male undergraduate students enrolled in the Harvard Alumni Health Study and failed to find a significant association between the total energy expended on physical activity in 1988 and the subsequent development of PD over 61,644 person-years follow-up. There was also no relationship between PD and the different physical activities, which included stair climbing, sports participation, leisure activity, or walking (Logroscino et al., 2006). Roos et al. (2018) investigated the hazard rate for developing PD based on BMI and sitting time in 41,638 participants from the Swedish National March Cohort. They failed to identify a significant association between developing PD and sitting time dichotomized into less than 6 h per day and 6 h or greater per day, using multivariable Cox proportional hazard models (HR = 1.06; 95% CI 0.76–1.47; Roos et al., 2018).

## Exercise and the Rate of PD Motor and Non-Motor Symptom Progression

Exercise may also be disease-modifying in fully manifest PD as well as in prodromal and preclinical stages. Longitudinal cohort studies have demonstrated that exercise can be a predictor of slower progression of both motor (parkinsonism, strength, balance, and gait) and non-motor [quality of life (QOL), cognitive function, anxiety, and depression] symptoms. In the population-based Parkinson’s Environment and Gene study in central California, Paul et al. evaluated the association between physical activity and progression of both motor and non-motor symptoms (NMS) in 244 subjects with early PD (within 3 years of diagnosis). They analyzed the subjects’ history of ever having participated in competitive sports and their overall physical activity level, in metabolic-equivalent hours per week (MET-h/week, a measure that incorporated duration and intensity of physical activity), across 4 age periods of adulthood. Over 5.3 years of follow-up they observed that those with a history of competitive sports were less likely to suffer a 4-point decline on the Mini-Mental State Exam (MMSE; HR = 0.46; 95% CI 0.22–0.96), or convert to stage 3 on the Hoehn and Yahr scale of motor disability (HR = 0.42; 95% CI 0.23–0.79). There was also a trend between higher MET-h/week (IQR) and slower progression on MMSE and conversion to Hoehn and Yahr stage 3, with HR 0.71 (95% CI 0.51–1.01), and 0.73 (95% CI 0.53–1.00), respectively, (Paul et al., 2019).

In the National Parkinson’s Foundation Quality Improvement Initiative (NPF-QII) Registry data, an observational prospective longitudinal study of PD subjects, Oguh et al. analyzed exercise habits in 4866 enrolled individuals. Their secondary aim explored the relationship between baseline exercise level (ordinally categorized into 3 groups by self-reported hours of exercise per week) and motor (“Timed Up and Go” test), QOL [Parkinson Disease Questionnaire-39 (PDQ-39)] and cognitive (verbal fluency and delayed 5-word recall) outcomes at 1-year follow-up in 2252 of these individuals. They found that “regular exercisers,” defined as participating in 2.5 h or more of exercise per week had better motor, QOL, and cognitive outcomes, compared to those in the “low exercisers” or “no exercisers” groups (Oguh et al., 2014). Subsequent to this study, Rafferty et al. evaluated exercise level and its association with the rate of change in motor and QOL outcomes over 3 visits (baseline, roughly 1-and 2-year time points) in a cohort of individuals from the NPF-QII. They observed that “regular exercisers” at baseline or those who became “regular exercisers” after the baseline visit, had a slower rate of decline in the motor and QOL outcomes over the 2-year period, compared to those who were not “regular exercisers,” or who became “regular exercisers” after the 2nd visit (1-year point; Rafferty et al., 2017). Both studies underline the potential importance of physical activity and, more specifically, meeting the recommended 2.5 h of exercise per week. Neither study assessed the mode or intensity of exercise.

Over a 2-year longitudinal cohort study, Combs-Miller and Moore (2019) sought to identify exercise behaviors (mode of exercise, minutes of exercise per week, and peak rate of perceived exertion) that could predict motor (grip strength, 10-meter walk test, functional reach test, and activities-specific balance confidence scale), and QOL (PDQ-39) outcomes in a convenience sample of PD subjects. In their models, a higher peak rate of perceived exertion predicted favorable motor and QOL outcomes, and it appeared to be a better predictor of outcomes than the previously studied “hours of exercise per week.” Mode of exercise was not included in their models as it was significantly related to peak rate of perceived exertion (Combs-Miller and Moore, 2019).

In the *de novo* (initially unmedicated) PD cohort of the Parkinson’s Progressive Markers Initiative (PPMI), Amara et al. investigated the relationship between scores on the Physical Activity Scale of the Elderly (PASE) and the progression of their motor and NMS over 2 years, from year 2 to year 4. They observed that higher PASE scores (i.e., greater physical activity) were associated with slower progression of motor symptoms, assessed by MDS-UPDRS part 3, MDS-UPDRS total (parts 1 to 3), and walking and balance scores; and with slower progression of NMS, assessed by semantic fluency, symbol digit modalities test, geriatric depression scale, state-trait anxiety inventory, and Montreal Cognitive Assessment (MoCA) over the 2-year period (Amara et al., 2019). Of note, serial imaging of the dopamine transporter was also assessed in the striata of these PPMI subjects between years 2 and 4, but showed no association with the preceding PASE scores (Amara et al., 2019). Thus, although these favorable associations between physical activity and subsequent clinical progression are consistent with a potentially protective effect on the underlying neurodegeneration, they may reflect alternative, non-causal mechanisms, including simply a symptomatic benefit, and like that seen with dopaminergic drug therapy.

## Basic Science Supporting Exercise as a Disease Modifier in PD

In animal models of PD, exercise has shown to be neuroprotective against the neurotoxins, 6-OHDA and MPTP. Several protective mechanisms have been implicated, including neurotrophic growth factors release, anti-oxidation, and anti-inflammation (Cotman et al., 2007; Monteiro-Junior et al., 2015). Tillerson et al. (2003) demonstrated in both 6-OHDA and MPTP rodent models that fixed-speed treadmill exercise (motivated by avoidance of weak electric shock at the back of the treadmill during training and subsequently by avoidance of bristles or a human hand behind them) twice a day for 10 days post-lesioning resulted in recovery of behavioral deficits and attenuated the loss of striatal dopamine, DOPAC, homovanillic acid, dopamine transporter, tyrosine hydroxylase, and vesicular monoamine transporter compared to those rodents who were not exposed to exercise. A prior study by Tillerson et al. (2001) also showed that forcing unilaterally 6-OHDA-lesioned mice to use their contralateral, impaired forelimb (by casting the ipsilateral, unaffected forelimb) for the first 7 days post-intrastriatal 6-OHDA infusion could also attenuate both the resulting neurochemical as well as behavioral deficits. They postulated that this neuroprotection was due to the release of neurotrophic growth factors, and then provided evidence for glia cell-derived neurotrophic factor (GDNF) as a candidate mediator (Cohen et al., 2003).

Later, Gerecke et al. demonstrated that voluntary exercise could also offer neuroprotection in toxin models of PD, arguing that increased physical activity, rather than stress, triggered the protection observed in the earlier experimental paradigms of forced or fear-motivated exercise. They also investigated the duration and intensity of activity that is warranted prior to lesioning in order to prevent neurodegeneration in the MPTP mouse model. They found that mice housed with unrestricted access to an exercise wheel over 3 months covered 7.5 km/day and this voluntary wheel running protected them from subsequent MPTP-induced neurotoxicity, with complete preservation of the midbrain dopaminergic neurons and partial attenuation of striatal dopamine loss observed in MPTP-lesioned control mice that had been housed without a wheel. They also nicely demonstrated that the exercise effect was (dose-dependent). A briefer duration of 2 months or a reduced intensity limited to 2/3 of the maximum voluntary rate (i.e., with the wheel locking daily after 4.8 km of running) conferred significant but less protection, whereas limiting the preceding running wheel access to only 1 month (without restriction on daily running distance) or to only 1/3 of the maximum voluntary rate (but for the full 3 month period) conferred no protection (Gerecke et al., 2010).

Hsueh et al. (2018) investigated the effects of voluntary running wheel exercise on gait, cognitive function, depression, and histology in 6-OHDA-lesioned rats. The rats assigned to the exercise group showed significantly decreased deficits of the motor and non-motor outcomes compared to the control group, and had increased expression of brain-derived neurotrophic factor (BDNF). Increased expression of BDNF and GDNF after exercise has also been demonstrated in MPTP mouse models (Palasz et al., 2019). Other studies have evaluated exercise in MPTP-treated mice heterozygous for the BDNF gene (Gerecke et al., 2012), or in 6-OHDA-lesioned rats treated with an inhibitor of BDNF receptor-induced intracellular signaling (Real et al., 2013). These studies suggest that BDNF receptor expression and function are required for exercise to confer protection in PD models. Data on other neurotrophic factors in PD models are limited, with the majority of data in healthy humans, or animal models (Neeper et al., 1996; Rasmussen et al., 2009; O’Callaghan et al., 2019).

In addition to neurotrophic factors, other candidate mediators of the potential neuroprotective effects of exercise have been explored. The Nrf2 antioxidant response pathway has also been implicated as such a candidate in local toxin [MPP+ (Tsou et al., 2015) and 6-OHDA (Aguiar et al., 2016)] models of PD in rodents, as has hypoxia inducible factor 1α (HIF1α) in an MPTP model (Smeyne et al., 2015). Koo et al. (2017) assessed α-synuclein expression and inflammatory responses following an 8-week exercise program in MPTP mouse models. They observed reduced α-synuclein expression and downregulation of the toll-like receptor 2 and its downstream inflammatory molecules following the exercise program. Tuon et al. also observed reduction in dopaminergic neurodegeneration and inflammation in their 6-OHDA mouse models following 8 weeks of exercise (strength training or aerobic training). They suggested that exercise stimulated Sirt1 activity, which resulted in modulation of neuroinflammation, and mitochondrial function (Tuon et al., 2015). Activity may also affect neuronal connectivity, and neurotransmission of dopamine and glutamate, reviewed by Petzinger et al., 2015. Lastly, exercise has been shown to protect against oxidative stress and mitochondrial dysfunction, reviewed by Svensson et al. (2015).

## Randomized Controlled Trials (RCTs) of Exercise and PD

There have been many clinical trials, systematic reviews and meta-analyses of exercise as a symptomatic therapy for PD motor and NMS (Grazina and Massano, 2013; Shu et al., 2014; Tomlinson et al., 2014; Lamotte et al., 2015; Mehrholz et al., 2015; Uhrbrand et al., 2015; Mak et al., 2017; Tang et al., 2019). Nevertheless, the variability in study design, exercise mode and regimen, participant selection, and outcome measurement tool used in the RCTs makes comparison between the studies difficult, and results in the inability to define “exercise prescription”; i.e., the formulation (mode of exercise), dose (intensity of exercise), route (home vs outpatient setting), frequency (daily vs weekly), and duration (short- vs long-term). While not intended to exhaustively cover all exercise RCTs in PD, this section summarizes some of the more recent and rigorous interventional study data, highlighting RCTs that addressed the important question of “exercise prescription” in their design. This section aims to lay the groundwork and underline the need for more rigorously designed exercise RCTs, as will be discussed in our final section “Designing clinical trials for exercise.”

Is any form of exercise sufficient for symptomatic benefit? Tango, treadmill running, resistance training, tai-chi, qigong, boxing, aqua therapy, different forms of dance, yoga, and multimodal regimens, are only some of the many exercise regimens that have been evaluated. In addition to the different modes of exercise studied, RCTs of exercise have focused on several different motor outcome measures including gait speed, freezing, balance, flexibility, endurance, cardiovascular fitness, motor strength in arms, legs and trunk, and motor symptoms on UPDRS assessment. Lauze et al. compiled the results and outcome measures in 106 physical activity intervention papers from 1981 to 2015, and found that 57.2% of the 530 different physical capacity outcome measures significantly improved after intervention (Lauzé et al., 2016). The potential for improvement was greater for upper and lower limbs, fair for flexibility and range of motion, and poor for trunk outcome measures. Tang et al. carried out a meta-analysis of 19 RCTs of exercise in PD and found that different modes and regimens of exercise were associated with different PD symptom benefits, ranging from improved gait velocity to lower UPDRS 3 motor scores, and faster TUG time and better QOL or balance. In their subsequent network meta-analysis, tango dance was the only mode of exercise to show benefit in these 5 outcomes of interest, and in the six-min walk test (Tang et al., 2019). Tango has been shown to be beneficial in most PD studies (Duncan and Earhart, 2012; Duncan and Earhart, 2014; McNeely et al., 2015). Other forms of patterned dance regimens like Waltz, Foxtrot and Irish set dancing have also reported positive effects on balance, functional mobility, motor impairment, and QOL in PD (Hackney and Earhart, 2009; Sharp and Hewitt, 2014; Shanahan et al., 2015).

Despite NMS of PD being more impactful than its classical motor symptoms on QOL (Martinez-Martin et al., 2011), they are often not the primary outcome measure of interest in trials of symptomatic and disease-modifying candidate therapies. There is a relative paucity of RCT data on exercise and NMS (Cusso et al., 2016; Subramanian, 2017). Nonetheless, the limited literature has suggested favorable effects of exercise on various NMS, with improvement in cognitive impairment and sleep (reviewed in Ahlskog (2018), Bhalsing et al. (2018), Stuckenschneider et al. (2019)), mood disorders (Wu et al., 2017), pain (Allen et al., 2015), and autonomic dysfunction (Kanegusuku et al., 2017). David et al. (2015) investigated cognitive function (Digit Span Forwards and Backwards, Stroop Color-Word Interference, and Brief Test of Attention) in the Progressive Resistance Exercise Training- PD (PRET-PD) trial. In this study, both subjects in the Progressive Resistance exercise group and those in the modified Fitness Counts exercise group were observed to improve on cognitive testing at 12 and 24 months, relative to their baseline assessment. There was no difference in the level of improvement between the two exercise groups (David et al., 2015). Although there was no control group, these results provide class IV evidence for exercise improving cognitive function, as cognition would be expected to decline over time due to aging and longer PD duration (Rabbitt et al., 2001; Muslimović et al., 2009). A meta-analysis of 9 RCTs evaluating cognitive function (assessed by PDQ-39, MoCA, and Trail Making Test) and different modes of exercise [tango dance, treadmill, cycling, combined cognitive training (Wii Fit) and motor training, and multimodal regimens] demonstrated improved or preserved cognition with exercise, with significant results in the tango group, treadmill, and combined cognitive training and motor training group (da Silva et al., 2018). This meta-analysis observed that treadmill training (high-intensity, three times per week, 60 min per week for 24 weeks) was associated with the largest effect size for cognitive improvement (effect size 1.3; Nadeau et al., 2014; da Silva et al., 2018).

This year, Amara et al. (2020) published their RCT of exercise, which assessed objective markers of sleep efficiency and architecture in individuals with PD. Using polysomnography data at baseline and post-intervention, they found that subjects undergoing supervised exercise, in the form of resistance training and body-weight functional mobility exercises, 3 times per week for 16 weeks, had improved sleep efficiency with greater total sleep time, less time awake after sleep onset and increased slow-wave sleep, compared to subjects in the control group (sleep hygiene, no exercise; Amara et al., 2020). Other exercise studies have also observed subjective improvements in sleep (Nascimento et al., 2014; Frazzitta et al., 2015; Silva-Batista et al., 2017).

In individuals with chronic illnesses, exercise can be as beneficial as medical therapies or cognitive behavioral therapy for treatment of depression (Blumenthal et al., 2007; Herring et al., 2012; Cooney et al., 2013). Systematic reviews of exercise RCTs and quasi-experimental studies for managing depression in PD have shown inconsistent results, with some studies showing improvement in depressive symptoms and others showing no change (Wu et al., 2017). A plausible explanation for these conflicting results is the different study methodology with differences ranging from depression outcome measurement tool used (Beck Depression Inventory; Hospital Anxiety and Depression Scale; Hamilton Rating Scale of Depression; Montgomery-Asperg Depression Rating Scale, The Geriatric Depression Scale; and Beck Anxiety Inventory) to exercise intervention applied. Da Lima et al. investigated the antidepressant effects of resistance training in individuals with PD. Using the 17-item Hamilton Depression Rating Scale, subjects in the resistance training exercise group (20 to 40 min of training, 2 days per week for 20 weeks) were observed to have a significant reduction in depressive symptoms, along with improved QOL, assessed on PDQ-39 and lower parkinsonism, assessed by total UPDRS, while the control group had no improvement in depressive symptoms, QOL or UPDRS after 20 weeks (de Lima et al., 2019).

Dysautonomia can cause a myriad of symptoms as the autonomic nervous system regulates gastrointestinal, genitourinary, thermoregulatory, and cardiovascular function. There is scarce trial data on exercise and PD autonomic function. Kanegusuku et al. examined cardiac autonomic modulation (sympathetic and parasympathetic nervous system) and cardiovascular responses to autonomic stress tests in PD subjects undergoing 12 weeks of progressive resistance training (PRT) compared to a control group. At 12 weeks, they observed reduced low frequency R-R interval variability, and less of a decrease in systolic blood pressure to orthostatic stress in the PRT group compared to controls. This improvement in sympathetic modulation and cardiovascular response to exercise in PD subjects suggests a potential therapeutic strategy for dysautonomia that requires further exploration (Kanegusuku et al., 2017).

Longitudinal cohort data suggests that regular exercise, defined as 2.5 h or greater per week, is associated with slower progression of motor and non-motor symptoms. However, the dose (i.e., intensity) of exercise to prescribe is currently unknown. Several recent studies have addressed intensity of exercise in PD (Shulman et al., 2013; Schenkman et al., 2018; van der Kolk et al., 2019). Shulman et al. randomized 67 subjects to one of three exercise arms: low-intensity treadmill exercise, high-intensity treadmill exercise, and stretching and resistance exercises in their single-blinded, single-center trial. At 3 months, they found that all groups improved on the six-min walk test with the greatest change in the low-intensity treadmill group. Both the treadmill exercise groups saw improvements in cardiovascular fitness, and only the stretching and resistance exercise group demonstrated increased muscle strength. Interestingly, these findings suggested that more intense exercise may not produce greater benefits, in contrast to other studies, as will be explored shortly. In addition, their results supported the concept that different modes of exercise produce different motor benefits, postulating that an “exercise prescription” should incorporate several modes of physical activity (Shulman et al., 2013).

Schenkman et al. also compared intensity of exercise in their phase 2, multicenter, single-blinded RCT entitled The Study in Parkinson Disease of Exercise (SPARX). Their study randomized 128 subjects to one of three arms: high-intensity treadmill (attaining HR 80–85% maximum heart rate), moderate-intensity treadmill (attaining HR 60–65% maximum heart rate), and a wait-list control group, representing usual care. At 6 months, the average worsening in UPDRS motor score was 0.3 points (95% CI -1.7–2.3) for high-intensity, 2.0 points (95% CI 0.3–3.7) for moderate-intensity, and 3.2 points (95% CI 1.4–5.1) for usual care group participants. The between-group difference in the high-intensity vs usual care included their *a priori* prespecified 3.5 points for futility. Additionally, there was a between-group difference in the high-intensity vs usual care in a metabolic measure of physical fitness (VO_2_ max change; *p* < 0.0001). Contrary to Shulman et al., their results suggested a potential benefit of high-intensity exercise over moderate-intensity in PD (Schenkman et al., 2018), and led to the initiation of a phase 3 RCT of this intervention with enrollment expected to begin by late 2020 (ClinicalTrials.gov, n.d.).

Lastly, Van der Kolk et al. compared home aerobic training to home stretching exercises using a web-based system, in their single-center, randomized, controlled, and putatively double-blinded trial entitled the Park-in-Shape trial. Following 6 months of exercise, the authors found a between-group difference of 4.2 points (95% CI 1.6–6.9), with less worsening on the motor scale in the aerobic training group (1.3 points) than in the stretching “active control” group (5.6 points). This between-group difference exceeded by 20% their *a priori* estimation of a minimum clinically relevant result, and was accompanied by a greater improvement in VO_2_ max change (*p* < 0.0001) among those randomized to aerobic exercise (van der Kolk et al., 2019). Similar to Schenkman et al., higher intensity exercise seems to be more beneficial in PD.

Recent RCTs of exercise in PD have incorporated health technology in their study design. As mentioned in the preceding paragraph, Van der Kolk et al. used a web-based system with virtual reality software containing gamified elements, a motivational app, and remote supervision, in their trial. Although, its application and utility compared to usual care were not assessed in this study, they plan to analyze it in the future, given its potential role in optimizing adherence and compliance (van der Kolk et al., 2019). Ellis et al. carried out a 12-month, single-blinded, pilot RCT comparing subjects who underwent exercise with a pedometer to subjects who underwent the same exercise program but with the assistance of a mobile health app and a pedometer. Although similar motor outcomes were seen in both groups, a subgroup analysis suggested additional benefit of the app in the more sedentary subjects (Ellis et al., 2019). Numerous other recent studies have also compared exergaming or virtual reality-based training with other exercise regimens or control groups, and found similar or better outcomes in the intervention group (Gandolfi et al., 2017; de Melo et al., 2018; Ferraz et al., 2018; Nuic et al., 2018).

The route (i.e., home vs. outpatient setting) of exercise is also unclear. The increasing use of virtual reality software and wearable sensors will allow people with PD to exercise at home or in otherwise decentralized, non-clinical settings. Flynn et al. carried out a meta-analysis on 16 trials of PD subjects, comparing balance-related function in subjects undergoing home-based exercises compared to either controls (*n* = 12) or center-based exercise programs (*n* = 4), over a 3 to 26-week period, with a median of 30.5 sessions. They found that balance-related activities in home-based programs were equivalent to center-based programs and better than usual care (Flynn et al., 2019), suggesting that home or local programs may be as effective as center-based, allowing patients to select their program preference.

## Designing Clinical Trials for Exercise

Epidemiological, basic science and randomized controlled trial (RCT) data provide overall compelling evidence that exercise in one form or another is beneficial and should be encouraged in individuals with PD. However, the precise “exercise prescription” that may be most effective overall or for a subset of patients remains to be established. The numerous trials to date have not optimized the prescription for several reasons. The most obvious impediment to comparison is the substantial variation in their exercise regimens (Silva et al., 2019). In addition, the small sample size in most of the studies, with a median of 40 subjects in total, often contributes to inadequate power and thus the predictable inability of studies to detect between-group differences (Silva et al., 2019). Trials to date have failed to differentiate symptomatic from disease-modifying effects of exercise due to the frequent use of parallel-group design along with the short duration and follow-up of these trials, often restricted to less than 5 months. Also concerning is the startlingly high rate of publication bias with only 26% of registered PD-exercise trials from 2000 to 2017 publishing their results in scientific journals. Similarly, there was also a high risk of bias with unblinded assessors of outcome in over half of the trials; and only 63% of trials had a control-comparator arm (Silva et al., 2019). While unblinding of research subjects to treatment assignment is difficult to avoid [though it can be minimized as attempted by van der Kolk et al. (2019)], there is little reason to allow unblinding of raters in an otherwise rigorously designed exercise trial. Currently, 80 PD-exercise studies are registered and listed as active on clinicaltrials.gov (ClinicalTrials.gov, n.d.). Although it is exciting to see ongoing research in this area, it is of critical importance that future studies are thoughtfully developed and rigorously conducted so that we can answer the fundamental question of “exercise prescription.”

Exercise trials present unique opportunities and challenges for investigators and subjects. From an individual subject standpoint, being involved in an exercise trial provides them with the chance to become more physically active, and develop a structured training plan with increased supervision and external motivation. The role of exercise in PD has attained great interest in both research and public forums (Park Found, n.d.). For investigators, this increased enthusiasm for exercise may translate into quicker subject recruitment and improved adherence to protocol. Moreover, these trials may be comparatively easy to initiate with less regulatory, intellectual property, and financial hurdles compared to trials investigating new pharmaceutical agents or medical devices. For all parties involved, these trials have lower safety concerns, with several studies demonstrating no or minimal morbidity in individuals with PD, as reviewed in Mak et al. (2017). Lastly, the increased utility of ehealth technology lends itself nicely to designing new exercise trials, as will be expanded upon below.

However, there remain several obstacles to exercise trials that need to be better addressed during the study design process. They range from blinding and compliance challenges, which are particularly problematic for exercise interventions, to symptomatic effects that may confound interpretation of long-term outcomes (as well known for dopaminergic drugs), to more universal problems of recruitment, retention, and outcome variability. Other unique challenges of exercise trials include a greater risk of selection bias as recruitment may be through convenience sampling, or the study may deliberately enrich for those subjects who are physically able and eager to participate in exercise, consequently reducing the generalizability of the results. The willingness to partake in exercise is also impacted by other factors including mood, energy, motivation, and self-efficacy levels (Ellis et al., 2011). Moreover, the best outcome to assess is often unclear as there are a variety of measurement tools available (Mak et al., 2017) and improvement in a metric may depend on the specific exercise regimen (e.g., BESTest for balance vs. six-min walk test for aerobic capacity.) Lastly, gender differences have been seen for exercise, with women responding differently than men (Chen et al., 2005; Roos et al., 2018). This difference has also been observed for other lifestyle risk factors in PD (Ascherio et al., 2001; Chen et al., 2002).

New strategies to overcome these design obstacles are now being pursued. For example, recent results from the above summarized independent SPARX (Schenkman et al., 2018) and Park-in-Shape (van der Kolk et al., 2019) trials, both well-designed phase 2 exercise studies, have converged in suggesting the greater benefit of high-intensity, aerobic exercise as a critical “prescription” element. On this foundation, a potentially more definitive, multi-site, phase 3 RCT has been developed by Corcos and colleagues (ClinicalTrials.gov, n.d.) with study start expected in 2020. For several reasons it may better distinguish disease-modifying from transient or symptomatic effects of a high-intensity treadmill intervention than have previous studies. SPARX3 is designed to enroll 370 participants with early, untreated PD and randomize them 1:1 to four weekly sessions of treadmill walking sessions each with 30 min at 80–85% maximum heart rate vs 60–65% maximum heart rate for 18 months, which is three times the duration of exercise in the earlier phase 2 studies of aerobic exercise (for 6 months). Second, the trial includes a final assessment after 24 months to gage any clinical effect’s persistence. Third, in addition to the primary, clinical outcome of MDS-UPDRS change at 12 months, a secondary, neuroimaging biomarker outcome of change in striatal dopamine transporter (DAT) binding signal is included at 12 months, and may suggest a structural basis of any demonstrated clinical benefit. If a third DAT scan were added after completion of the intervention at 21–24 months, it may help substantiate a persistent protective effect of exercise on the dopaminergic innervation of the striatum.

Additional obstacles may be overcome using strategies emerging from advances in mobile technologies, leading to novel design opportunities for exercise trials in the not too distant future. For example, one can envision a large-scale, long-term RCT of exercise in PD with subjects fully engaging in the study through a single, self-contained application on a mobile phone or wearable device. The app could house or integrate all trial elements from e-consent process, to neurologist-interfaced diagnostic certification, to randomized exercise program assignment, and titration algorithms akin to drug dose adjustment driven by targeted blood biomarker levels as in the SURE-PD trial (Parkinson Study Group SURE-PD Investigators et al., 2014) with blind-preserving instructions to maintain or increase exercise levels to achieve an assigned target (e.g., 10, 20, 40, and 60%, etc. of baseline activity) of which the participant is not specifically aware. Moreover it could monitor activity (e.g., step count), and contain tailored motivational features built on game theory and bolstered by social network engagement or centralized trainers and clinicians [with proof-of-concept established in the recent Park-in-Shape study (van der Kolk et al., 2019)], and finally to study outcome metrics for PD progression as captured for example by the PD-tailored mPower study app (Bot et al., 2016), or Roche PD Mobile Application (Lipsmeier et al., 2018). These outcome metrics may include objective measures of motor and non-motor functions like finger tapping speed and memory (which are actively collected through scheduled tasks) or passively collected mobility and socialization data, as well as patient reported outcomes and possibly physiological assessments of physical fitness [e.g., VO_2_ max (Sage Bionetworks, n.d.)]. Such a trial has the potential to be entirely decentralized permitting global implementation across language, socioeconomic and geographic boundaries to a far greater extent than traditional neurology clinic-centered trials, and thereby allowing for a plausibly large sample size (e.g., in excess of 10,000 participants with PD) that could offset amplified limitations of compliance, retention and outcome variability.

## Conclusion

There is increased recognition of the potential for long-term benefits of exercise in individuals with PD. In this review article, we summarized the supportive epidemiological, basic science, and randomized control trial data on exercise in PD. However, many outstanding questions remain, including whether exercise is truly neuroprotective and disease-modifying versus symptomatically but reversibly beneficial, and if so, what exercise should we recommend. Rigorously designed, large-scale studies are warranted. Innovative motivational methods and outcome assessments, adapted from emerging digital health technologies, will enhance the potential for such trials to answer these important questions.

## Author Contributions

GC and MS were involved with the concept and design of the manuscript. GC drafted the manuscript. GC and MS revised the manuscript and gave final approval on the submitted manuscript. Both authors contributed to the article and approved the submitted version.

## Conflict of Interest

The authors declare that the research was conducted in the absence of any commercial or financial relationships that could be construed as a potential conflict of interest.
